# Targeting subchondral osteoporosis in osteoarthritis: biomechanical crosstalk and the therapeutic rationale for inflammatory microenvironment remodeling

**DOI:** 10.3389/fimmu.2026.1944298

**Published:** 2026-09-04

**Authors:** Mingli Han, Longfei Han, Suya Zhang, Long Tian, Guifeng Luo, Kun Lin, Hai Su, Junjiao Zhang, Fan Yang, Mincong He, Qiushi Wei

**Affiliations:** 1 Guangzhou University of Chinese Medicine, Guangzhou, China; 2 Shenzhen Pingle Orthopedic Hospital (Shenzhen Pingshan District Hospital of Traditional Chinese Medicine), Shenzhen, China

**Keywords:** biomechanics, cellular senescence, immune microenvironment, macrophage polarization, osteoarthritis, osteoporosis

## Abstract

Current osteoarthritis (OA) management remains largely symptom-directed, while the contribution of osteoporosis (OP)-related and OA-associated subchondral remodeling to disease heterogeneity remains uncertain. This review integrates macroscopic load redistribution and lower-limb malalignment with local osteoimmune processes, including macrophage-state heterogeneity, Th17/Treg imbalance, senescence-associated secretory signaling, and bone–vascular coupling. We propose a bidirectional, phenotype-dependent framework in which abnormal loading may initiate compartment-specific subchondral remodeling, whereas high-turnover bone loss may reduce trabecular competence and amplify stress concentration; late OA may instead exhibit a low-turnover sclerotic phenotype. Existing evidence supports biological links among bone remodeling, inflammatory signaling, and osteochondral crosstalk but does not establish that anti-osteoporotic therapy reverses malalignment or consistently modifies OA progression. Bone-targeting treatment should therefore be considered a hypothesis requiring turnover- and phenotype-stratified clinical evaluation. The proposed time-window model is intended to organize future studies rather than provide current treatment recommendations.

## 1 Introduction

Osteoarthritis (OA) and osteoporosis (OP) are highly correlated degenerative diseases in the middle-aged and elderly population, and their comorbid processes involve precisely regulated pathophysiological stages such as bone loss, cartilage degeneration, and subchondral bone remodeling (1, 2). These steps often involve the involvement of multiple cell types, including osteoblasts, osteoclasts, chondrocytes, and immune cells. However, the synergistic deterioration of OA and OP has become a major challenge in orthopedic treatment under pathological conditions such as aging, menopause, and complex biomechanical changes (3). Despite the traditional view that OA and OP have antagonistic effects based on differences in bone mineral density, there is still a lot of clinical and epidemiological evidence that OA and OP present a very high comorbidity rate in elderly patients. Current treatments, such as non-steroidal anti-inflammatory drugs and chondroprotective agents, have had some success in alleviating the initial symptoms; however, many patients still face the challenge of continuous progression of the disease course, which is a challenge for the management of the disease, eventually moving towards prosthetic joint replacement. Therefore, it has become an urgent need to explore therapeutic targets from a new perspective of subchondral bone microstructure and mechanical conduction.

Traditionally, studies targeting osteoarthritis have typically focused on the degradation of the articular cartilage matrix and the inflammatory microenvironment, with subchondral bone serving as the mechanical supporting core of the joint, and subchondral bone serving as a structural support for the joint, plays a crucial role in osteoporotic-arthritis comorbidity (1, 4). In the early stages of the disease, local or systemic osteoporosis leads to a loss of bone mass in the subchondral bone region of the tibial plateau (which often appears as a low-density shadow on imaging), systemic osteoporosis not only exists as an independent systemic degenerative factor, but also is a ‘powerful catalyst’ for local joint mechanical abnormalities and subchondral bone degeneration caused by synovitis microenvironment, forming a vicious circle. The destruction of these microstructures subsequently translates into macroscopic biomechanical imbalance, driving subchondral bone micro-collapse and medial offset of the lower limb mechanical axis (i.e., straight leg flexion or genu varus deformity) (5). Although medial compartment knee osteoarthritis with varus deformity is the most classical and intuitive clinical model to explain this biomechanical imbalance, it is difficult to explain the mechanism of varus deformity. However, the core mechanism of “microstructural damage leads to abnormal macroscopic mechanical distribution” is also applicable to other types of load-bearing or non-load-bearing joints (such as the displacement of the center of stress of the hip joint, the degeneration and instability of the spinal facet joint, etc.). In recent years, breakthroughs in bone metabolism and biomechanics have shown that the structural degeneration of subchondral bone is closely related to a variety of intra-articular pathological changes. Multiple bone metabolic pathways, including RANKL/RANK/OPG axis, the Wnt/β-catenin signaling pathway, in addition to regulating bone homeostasis, dynamically control cartilage degradation and synovial inflammation through abnormal stress concentration. Despite the emerging recognition of subchondral bone as a key pathological hub, current narrative overviews often treat subchondral bone alterations either as localized features of primary osteoarthritis or focus extensively on biological cell fate plasticity across isolated cell lineages (6, 7). A recent comprehensive review by Zhang et al. (2026) elegantly highlighted how subchondral microenvironmental factors drive multi-lineage cell fate reprogramming in OP-OA comorbidities. However, a critical scientific and translational gap remains: how to seamlessly integrate macroscopic biomechanical axis decompensation (such as genu varus malalignment) with microscopic osteoimmune-metabolic networks (e.g., PIEZO1/FABP4/Type H vessel crosstalk), and translate these insights into a clinically actionable, temporally staged intervention framework (8).

This review aims to provide a framework for understanding the critical role of osteoporosis and subchondral bone in the progression of osteoarthritis, clearly elucidate their mechanical and pathological properties, and detail how the comorbid process is triggered by microenvironmental signals, and provide a theoretical basis for the prevention and treatment of osteoarthritis, such as abnormal mechanical stress concentration, metabolic imbalance of osteoclasts/osteoblasts, and high non-regeneration of cartilage (1, 2). Furthermore, we summarize current potential approaches to anti-osteoporosis treatment during OA comorbid with OP and highlight intervention strategies to delay arthritis by stabilizing the bone base. Finally, we discuss the main obstacles and future directions for targeted bone metabolism interventions in the comorbid field.

### 1.1 Literature search strategy

This narrative review was informed by a structured literature search of PubMed/MEDLINE, Web of Science Core Collection, and Embase for English-language articles published between January 1, 2000 and July 31, 2026. The search strategy combined controlled vocabulary and free-text terms related to the principal concepts of this review, including “osteoarthritis,” “osteoporosis,” “subchondral bone,” “bone remodeling,” “biomechanics,” “mechanical loading,” “malalignment,” “osteoclast,” “osteoblast,” “osteoimmunology,” “RANK/RANKL/OPG,” “macrophage polarization,” “cellular senescence,” “angiogenesis,” “type H vessels,” and “mitochondrial dysfunction.” A representative search structure was: (“osteoarthritis” OR “OA”) AND (“osteoporosis” OR “low bone mass”) AND (“subchondral bone” OR “bone remodeling” OR “biomechanics” OR “osteoclast” OR “osteoblast” OR “osteoimmunology”). Search terms and syntax were adapted to the requirements of each database. The reference lists and forward citations of key articles and relevant reviews were also manually examined to identify additional eligible studies.

Eligible publications were peer-reviewed studies addressing OA–OP comorbidity or mechanisms directly relevant to subchondral bone remodeling, biomechanical dysregulation, osteoimmune signaling, or bone–cartilage crosstalk. Evidence was prioritized in the following order: primary human OA–OP studies and well-characterized human OA cohorts; randomized or controlled clinical trials; prospective imaging, biomarker, and histomorphometric studies; high-quality systematic reviews, meta-analyses, and consensus statements; and mechanistic animal or *in vitro* studies when direct human evidence was unavailable. Duplicate publications, case reports, non-peer-reviewed articles, and studies that did not directly substantiate the statement under consideration were excluded. Studies centered on rheumatoid arthritis, temporomandibular joint OA, isolated systemic osteoporosis, herbal interventions, gut microbiota, or other indirect disease models were not used as direct supporting evidence unless they addressed a clearly transferable mechanism for which OA–OP-specific evidence was unavailable. In such cases, their indirect or hypothesis-generating nature was explicitly acknowledged.

Following study selection, citations were audited statement by statement for relevance, study population, disease model, intervention, outcome, and level of evidence. References that did not directly support the associated claims were removed or replaced, with preference given to primary OA–OP studies, human cohorts, randomized trials, and high-quality systematic reviews. Where direct OA–OP evidence remained unavailable, the limitation and extrapolative nature of the supporting evidence were stated in the text. Because the objective was an evidence-informed narrative synthesis rather than quantitative effect estimation, no formal meta-analysis was performed.

## 2 Pathological cascade of osteoporosis-osteoarthritis comorbidity and the central regulatory role of subchondral bone

**Figure 1:**
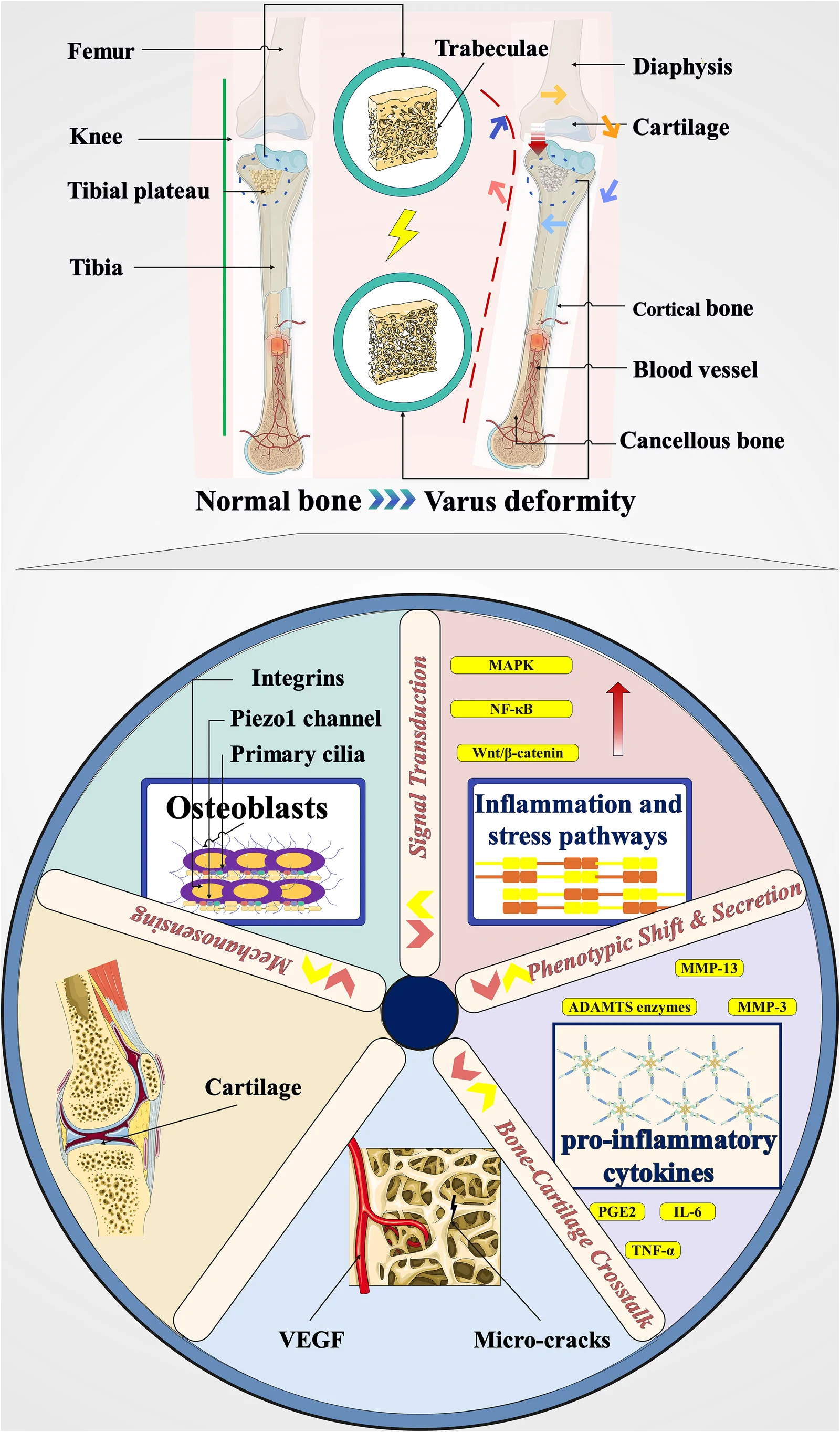
Pathological cascade of osteoporosis-osteoarthritis (OP-OA) comorbidity and the “mechano-biological” regulatory mechanism of subchondral bone. Path arrows indicate a proposed bidirectional model; evidence strength is heterogeneous.

The comorbid progression of osteoporosis and osteoarthritis is not a simple superposition of two independent degenerative lesions, but is caused by the loss of bone structural integrity, the characteristic pathological cascade of sequential breakdown of mechanical homeostasis and biological regulatory networks. As shown in the upper half of **Figure 1**, this pathological process presents a clear multidimensional spatio-temporal evolution profile, covering the full hierarchy of pathological alterations that shift from the line of force of the macroscopic lower limb to cross-cellular molecular crosstalk within the microenvironment, and the underlying mechanisms that underlie this process, based on the core differences in lesion progression (9).The development of comorbidity can be divided into two consecutive key stages. It is crucial to emphasize that this pathological progression is non-linear and highly heterogeneous. The uncoupling phase of bone metabolism occurs most frequently in postmenopausal and elderly people (10). The pathological features are abnormal hyperactivation of osteoclasts, and the rate of local bone resorption significantly exceeds the efficiency of bone formation mediated by osteogenesis (11, 12). Finally, it leads to progressive thinning and discontinuity of trabecular bone in the tibial plateau, which is characterized by local bone mineral density reduction on imaging, and then transitions to clinically detectable dominant mechanical decompensation, as the inherent biomechanical strength of subchondral bone continues to decline, it cannot compensate for the weight-bearing load of daily physiological activities, and diffuse micro-collapse occurs (13). The disintegration of the local structure of the subchondral bone directly induces the biological force line of the lower limb to shift to the medial side of the joint, which further triggers the progressive narrowing of the secondary joint space and the severe wear of the articular cartilage.

This review articulates a bidirectional crosstalk model between mechanical axis deviation and subchondral bone metabolism, rather than a strictly unidirectional sequence (**Figure 1** center schematic). The onset of comorbidity initially begins with the imbalance between microdamage and structural remodeling of subchondral bone trabeculae, a pathological event that can instantly break the original uniform stress distribution pattern in the joint microenvironment (14). Subchondral bone drives the progression of osteoarthritis exponentially through a dual synergistic mechanism of “macrostructural deformation” and “micromolecular crosstalk”. Notably, subchondral bone exhibits typical ‘temporal biphasic’ changes during OA-OP comorbid progression (15). In the early stage of the disease, osteoclast-dominated bone resorption is dominated by local osteoporosis and microstructural loss; the disease enters a late compensatory stage. At this point, resident subchondral osteoblasts are induced to transform into a pathological ‘sclerotic phenotype’ under abnormal mechanical perception in response to supraphysiological loading. At the transcriptional and molecular level, this phenotypic shift is driven by mechanical overload-induced hyperactivation of master osteogenic transcription factors, including Runx2 (Runt-related transcription factor 2) and Osterix (Osx/Sp7), via mechanosensitive pathways such as PIEZO1/MAPK and NF-κB (16). Concurrently, altered canonical Wnt/β-catenin signaling and elevated non-canonical Wnt ligands (e.g., Wnt5a) drive aberrant osteogenic lineage commitment. Paradoxically, these pathologically sclerotic osteoblasts oversecrete Wnt pathway inhibitors such as Sclerostin (encoded by SOST) and Dickkopf-1 (DKK1), alongside heightened expression of matrix mineralizers like Osteopontin (OPN/SPP1) and Osteocalcin (OCN) (17). Although this late-stage sclerosis presents as localized bone sclerosis with increased mineral density on imaging, the newly formed bone exhibits disrupted hydroxyapatite crystallization, poor structural mineralization, and diminished biomechanical elasticity (18). Thus, it represents a maladaptive pathological compensation that fails to cushion joint loads and instead accelerates upper cartilage wear. It not only fails to restore the normal mechanical buffering function, but instead exacerbates the wear of the upper cartilage, and the micro-collapse at the macro-level directly reshapes the force line distribution of the lower limb, which can be used as a reference for the design and construction of the lower limb. The abnormally elevated mechanical stress at the microscopic level deeply reconstructs the biochemical characteristics of the local microenvironment of the subchondral bone through conserved mechanical conduction pathways. When local biomechanical alterations, such as a typical knee varus deformity, induce a stress concentration beyond the physiological threshold in a specific joint chamber, the complete pathological cascade will be progressively advanced along sequential logic, and the pathological cascade will become more complex, as shown in the lower half of **Figure 1** (19). Osteoblasts first enter the mechanoperception stage through multiple classes of mechanoreceptors expressed on the surface, including PIEZO1 ion channels, integrin adhesion networks, and primary ciliary structures, which are involved in the mechanoperception of osteoblasts to accurately identify abnormally elevated mechanical load signals (20, 21). Non-physiological physical stimuli are then rapidly converted into downstream biochemical signals within the cell, and to identify the underlying mechanisms, induction of excessive activation of stress- and inflammation-related pathways such as MAPK, NF-κB, and Wnt/β-catenin ultimately forces resident osteoblasts to convert to a pathological “sclerosing phenotype”. Immediately following phenotypic switching, a global remodeling of the cell secretion profile occurs, with abnormally stressed osteoblasts, together with a damaged extracellular matrix, releasing large amounts of pro-inflammatory mediators such as IL-6, TNF-α, PGE2, etc. Also, over-expressed MMP-3, MMP-13, ADAMTS family, and other matrix-degrading proteases (22–25), more critically, pathological stress-induced micro-fissures in subchondral bone plates, superimposed VEGF-mediated neovascularization penetrating tidal line structure, and increased the expression of MMP-3, MMP-13, ADAMTS family, and other matrix-degrading proteases. It provides a physical channel for the above-mentioned destructive factors to penetrate directly into the cartilage layer (see **Figure 1** ‘Osteochondral Crosstalk’ module). Inflammatory factors and degrading enzymes originating from pathologically altered subchondral bone diffuse upward to the originally avascular hyaline cartilage layer through the “Osteochondral crosstalk” pathway.

This “inflammatory cascade” originating from abnormally stressed subchondral bone not only directly inhibits the anabolic activity of chondrocytes, but furthermore, the two major cartilage core structural proteins, type II collagen and aggrecan, are directly targeted and degraded by the “matrix-degrading enzymes” effect (26). With the progressive aggravation of the knee varus deformity, the peak contact pressure of the medial joint chamber increases sharply, which eventually drives the disease from a simple bone loss state to an irreversible destruction of articular cartilage (27, 28). Because the adult articular cartilage belongs to highly differentiated avascular tissue, the intrinsic regeneration ability is extremely limited, and the double blow of structural instability and molecular degradation eventually leads to full-thickness denudation of the cartilage layer and osteophyte formation at the joint edge (29). It is worth emphasizing that osteoporosis does not only play a role as an initial initiating factor of comorbid diseases, but also mediates the dynamic changes in bone mass and biomechanical properties, which may contribute to the development of osteoporosis (30). It is the core pathological regulation hub throughout the whole course of osteoarthritis deterioration, which will be further explained in subsequent chapters. Under the microenvironmental characteristics of limited inherent regeneration ability of adult cartilage, the complete molecular regulatory network and clinical translational potential of osteoarthritis treatment are realized by stabilizing the subchondral bone mechanical base with anti-osteoporosis intervention as the entry point.

Above; macromechanical decompensation and structural change: evolution from normal bone to varus deformity. Osteoporosis leads to progressive thinning and loss of the trabeculae below the tibial plateau, which reduces the mechanical strength of the subchondral bone (31). Under physiological load, structural collapse leads to micro-collapse, which causes the force line of the lower limb to move inward and the knee joint to Varus, which directly aggravates the abnormal compression and serious wear of local cartilage (32). lower panel: ‘mechano-biological’ molecular crosstalk within the subchondral bone microenvironment: pathological cascades triggered at the microscopic level by abnormally elevated mechanical stress. Mechanosensing: resident osteoblasts accurately recognize abnormal mechanical loading through Integrins, PIEZO1 ion channels, and primary cilia on their surfaces (33, 34). Signal Transduction: non-physiological physical stimuli are converted into intracellular biochemical signals that over-activate inflammatory and stress-related pathways such as MAPK, NF-κB, and Wnt/β-catenin. Phenotypic Shift & Secretion: the conversion of osteoblasts to a pathological “sclerosing phenotype” also releases large amounts of proinflammatory cytokines (e.g., PGE2, IL-6, TNF-α) and matrix-degrading proteases (e.g., MMP-13, MMP-3, ADAMTS enzymes) (35). Bone-cartilage Crosstalk: Micro-cracks created by subchondral bone plates superpose VEGF-mediated neovascularization to penetrate the tidal line, providing a physical channel for these destructive factors, drives an “persistent inflammatory signaling “ that spreads upward into the cartilage layer, accelerating irreversible degradation of the cartilage matrix (36, 37).

## 3 Cellular multidimensional interactions and factor cascade networks in the comorbid microenvironment

**Figure 2:**
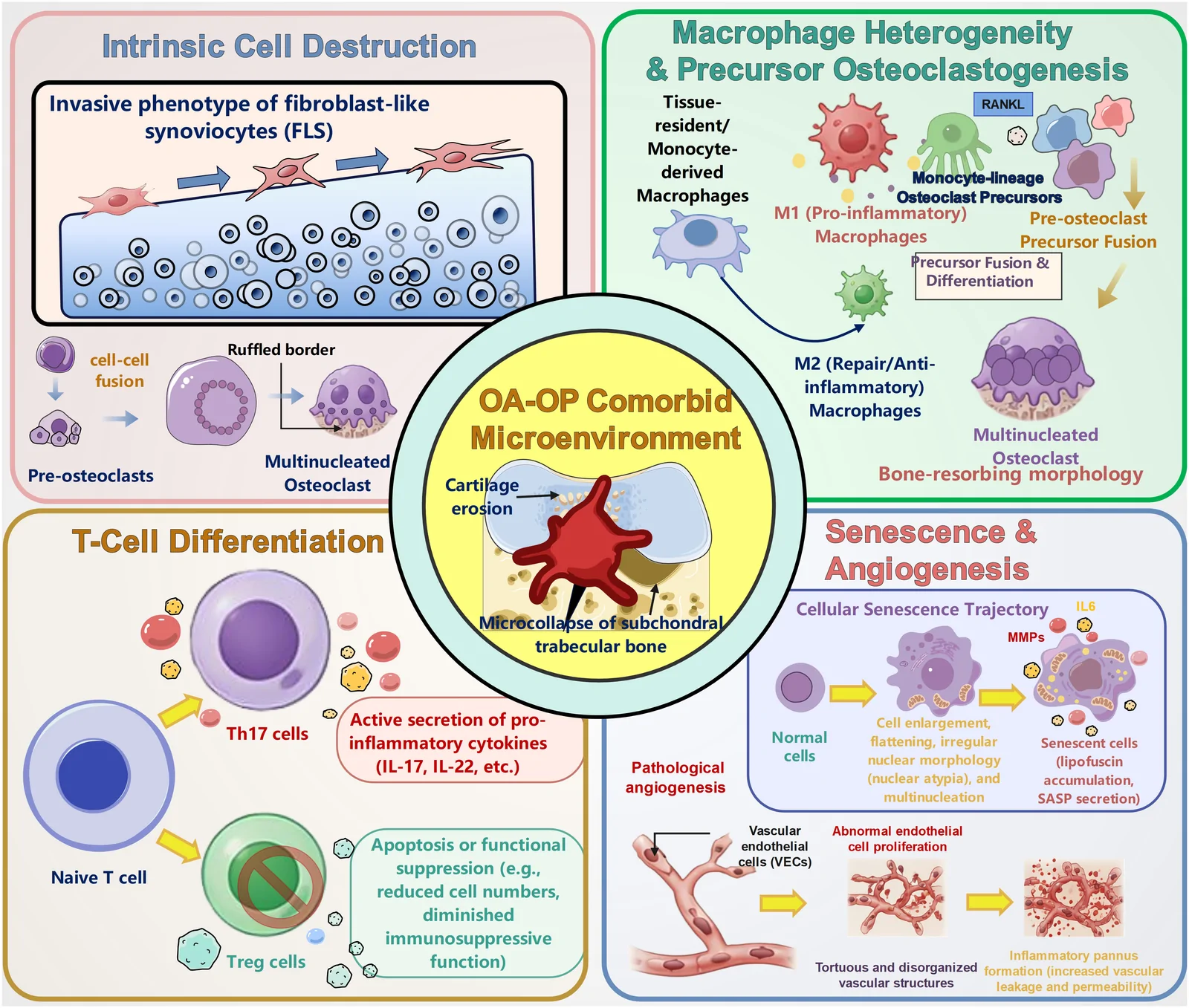
Multi-lineage cell interactions and pathological cascade networks in the osteoarthritis-osteoporosis (OA-OP) comorbid microenvironment.

The comorbid evolution of osteoarthritis (OA) and osteoporosis (OP) is far from the passive physical depletion caused by simple mechanical stress; rather, it is a cascade of deterioration triggered by local biomechanical remodeling, with multi-lineage cells deeply intertwined and accompanied by global imbalances in molecular regulatory networks (38). The traditional “Single cell theory” research perspective often sees only the trees but not the forest, and it is difficult to reveal the deep pathological logic under the co-disease microenvironment (39). In fact, it is the intricate crosstalk and functional linkage between cells of different lineages that construct the driving force of the core pathological axis of “Bone loss, force line deviation, and cartilage degradation”; a self-reinforcing positive feedback network of inflammation and tissue destruction is woven into the local area of the joint (**Figure 2**, Global Overview) (40, 41). As shown in **Table 1**, these different lineages of innate and immune cells play intricate but highly synergistic pathogenic roles in the microenvironment.

**Table 1:** Multi-lineage key cells and their pathogenic network roles in the OA-OP comorbid microenvironment.

Cell type	Role	References
Synovial fibroblasts	Secretion of RANKL promotes osteoclast differentiation; production of inflammatory factors (such as IL-6, TNF-α) drives synovitis and bone destruction; and it is a central effector cell of joint destruction	(51, 56, 60, 61)
Osteoclasts	It mediates bone erosion, periarticular bone loss, and systemic osteoporosis, and its overactivation drives subchondral trabecular bone loss in OA	(47, 49, 55, 60, 62)
T cells (Th17/Treg)	Th17 cells secrete IL-17 to promote inflammation and osteoclastogenesis; Treg cell functional imbalance exacerbates immune inflammation and bone destruction	(5, 58, 59)
Macrophages	Polarization imbalance (M1 pro-inflammatory/M2 anti-inflammatory) affects the joint inflammatory microenvironment; monocyte-macrophage precursors undergo multinucleated osteoclastogenesis under RANKL/CSF-1 stimulation to participate in bone resorption	(53, 55, 60, 63)
Monocyte-macrophage system	It affects inflammation and bone remodeling through epigenetic regulation (histone acetylation/deacetylation); it is an important bridge connecting immunity and bone metabolism	(26, 63)
The vascular endothelium	It participates in pannus formation in inflammation and affects bone formation and resorption by secreting angiogenic factors and regulating bone vascular coupling	(44, 52, 64–66)
Senescent cells	Accumulation in OA, secretion of SASP (senescence-associated secretory phenotype) factors, driving chronic inflammation and bone matrix degradation	(67–70)

### 3.1 Structural degradation and FLS-immune-vascular coupling at the osseous interface

In the osteoarthritic joint, tissue-resident parenchymal cells act in intimate concert with localized immune networks rather than as isolated physical units. Synovial fibroblasts (OA-FLS) serve as crucial immunomodulatory effectors bridging synovial inflammation with subchondral bone loss. When subjected to abnormal biomechanical stretch, OA-FLS adopt a pro-inflammatory secretory profile, releasing substantial quantities of RANKL alongside key monocyte chemokines such as CXCL12 and CCL2.Crucially, emerging evidence indicates that pathological micro-vessels breaching the subchondral tidemark barrier exhibit specific Type H endothelial markers (CD31^hi^Emcn^hi^). Under physiological conditions, Type H vessels strictly couple osteogenesis with angiogenesis via PDGF-BB/VEGF secretion. In the aberrantly stressed OA-OP microenvironment, however, excessive mechanical strain and FLS-derived chemokines drive dysregulated Type H vascular sprouting across the calcified cartilage interface (42, 43). These tidemark-penetrating Type H vessels act as direct “humoral and cellular conduits,” actively transporting subchondral inflammatory mediators (TNF-α, IL-6, MMP-13) and recruiting monocyte-macrophage precursors directly into the hyaline cartilage layer, bringing vessel-FLS-immune cell crosstalk to the forefront of subchondral osteoimmune degradation (44, 45).

### 3.2 The osteoimmune core: macrophage heterogeneity, T-cell imbalance, and SASP-driven remodeling

Rather than functioning via a passive physical response, the subchondral bone microenvironment during OA-OP comorbidity is governed by an intricate osteoimmune cascade. Macrophage Transcriptomic States and Precursor Osteoclastogenesis: Recent human OA single-cell RNA sequencing (scRNA-seq) and spatial transcriptomics studies have dismantled the classical binary M1/M2 macrophage paradigm, revealing a highly dynamic transcriptomic spectrum of joint-resident macrophages (46). Under localized subchondral stress, tissue-resident macrophage subsets undergo phenotypic reprogramming toward inflammatory, S100A8/A9-expressing subsets. More importantly, monocyte-macrophage precursors localized within subchondral marrow niches do not merely secrete pro-inflammatory cytokines; under the synergistic stimulation of osteoblast/FLS-derived RANKL and CSF-1, these precursor lineages undergo cell-cell fusion and multinucleation, giving rise to functional, bone-resorbing osteoclasts specifically concentrated at subchondral micro-cracks and trabecular resorption lacunae (47–49).OA-Specific Th17/Treg Imbalance: Adaptive osteoimmune regulation further amplifies this local destructive loop. In patients with knee OA, an elevated local Th17/Treg ratio in subchondral bone marrow and synovial tissues correlates strongly with joint pain and disease progression. Polarized pro-inflammatory Th17 cells actively secrete IL-17 and IL-22 within the joint microenvironment (50). IL-17 acts as a potent osteoimmune mediator that directly upregulates RANKL expression in resident osteoblasts and FLS, while simultaneously enhancing osteoclast precursor sensitivity to RANKL. Concurrently, regulatory T cells (Tregs), which normally maintain bone immune tolerance by secreting IL-10 and TGF-β to suppress osteoclastogenesis, suffer from numerical depletion and functional exhaustion in aging and stressed joint niches. This collapse of Treg-mediated immunosuppression allows unchecked osteoclastic bone resorption and accelerates subchondral trabecular thinning. SASP-Mediated Osteoimmune Remodeling: Superimposed on immune subset dysregulation is the accumulation of senescent osteocytes, osteoblasts, and chondrocytes within the subchondral bone. These senescent cells express a potent Senescence-Associated Secretory Phenotype (SASP), characterized by the continuous release of IL-6, IL-1β, TNF-α, MMP-3, and MMP-13. Within the subchondral niche, SASP factors operate as chronic local osteoimmune disruptors: upward, they suppress osteoblast osteogenic differentiation and RUNX2 expression; downward, they amplify local vascular permeability and attract osteoclast precursors. This SASP-driven osteoimmune feedback loop fuels persistent subchondral bone loss and microstructural collapse, driving the progression of OA-OP comorbidity.

OA-OP Comorbid Microenvironment: reveals the core structural features of the Comorbid Microenvironment, namely cartilage erosion and microcollapse of subchondral trabecular bone. Top left: Intrinsic Cell Destruction: osteoclast precursor cells fuse to form multinucleated osteoclasts with ruffled borders, which guide bone resorption; fibroblast-like synoviocytes (FLS) exhibit an aggressive phenotype (51, 52). Top right: Macrophage Heterogeneity & Precursor Osteoclastogenesis: depicting heterogeneous macrophage transcriptional states across a phenotypic spectrum rather than a strict M1/M2 dichotomy, alongside monocyte-macrophage progenitor fusion into osteoclast-like cells (53–55). Under RANKL and CSF-1 stimulation, monocyte–macrophage lineage precursors undergo osteoclastic differentiation, cell–cell fusion, and multinucleation to generate functional bone-resorbing osteoclasts. This process should not be interpreted as direct trans Bottom right: Senescence & Angiogenesis: the evolution of normal cells to a senescent phenotype (characterized by nuclear atypia, lipofuscin accumulation, and SASP factor secretion, e.g., IL-6, IL-1β, MMP-3, MMP-13); abnormal proliferation of vascular endothelium (VECS); and the formation of a tortuous and disordered inflammatory pannus, resulting in increased vascular permeability (56, 57). Bottom left: T-Cell Differentiation: initial T cells undergo an imbalance of differentiation, with pro-inflammatory Th17 cells being activated and secreting large amounts of factors such as IL-17 and IL-22, while Treg cells with immunosuppressive functions are reduced in number, undergo apoptosis, or are functionally suppressed (58, 59).

This table summarizes the core cell communities involved in the comorbid progression of osteoarthritis (OA) and osteoporosis (OP). Synovial fibroblasts (FLS) versus osteoclasts dominate the substantial destruction of joint architecture; polarization imbalance of macrophages versus T cells (Th17/Treg) drives a sustained inflammatory cascade; the vascular endothelium and senescent cells participate in the formation of inflammatory pannus and matrix degradation, which together build a self-reinforcing positive feedback network of tissue destruction (66).

## 4 The deep relationship between abnormal bone metabolism and markers of arthritis

**Figure 3:**
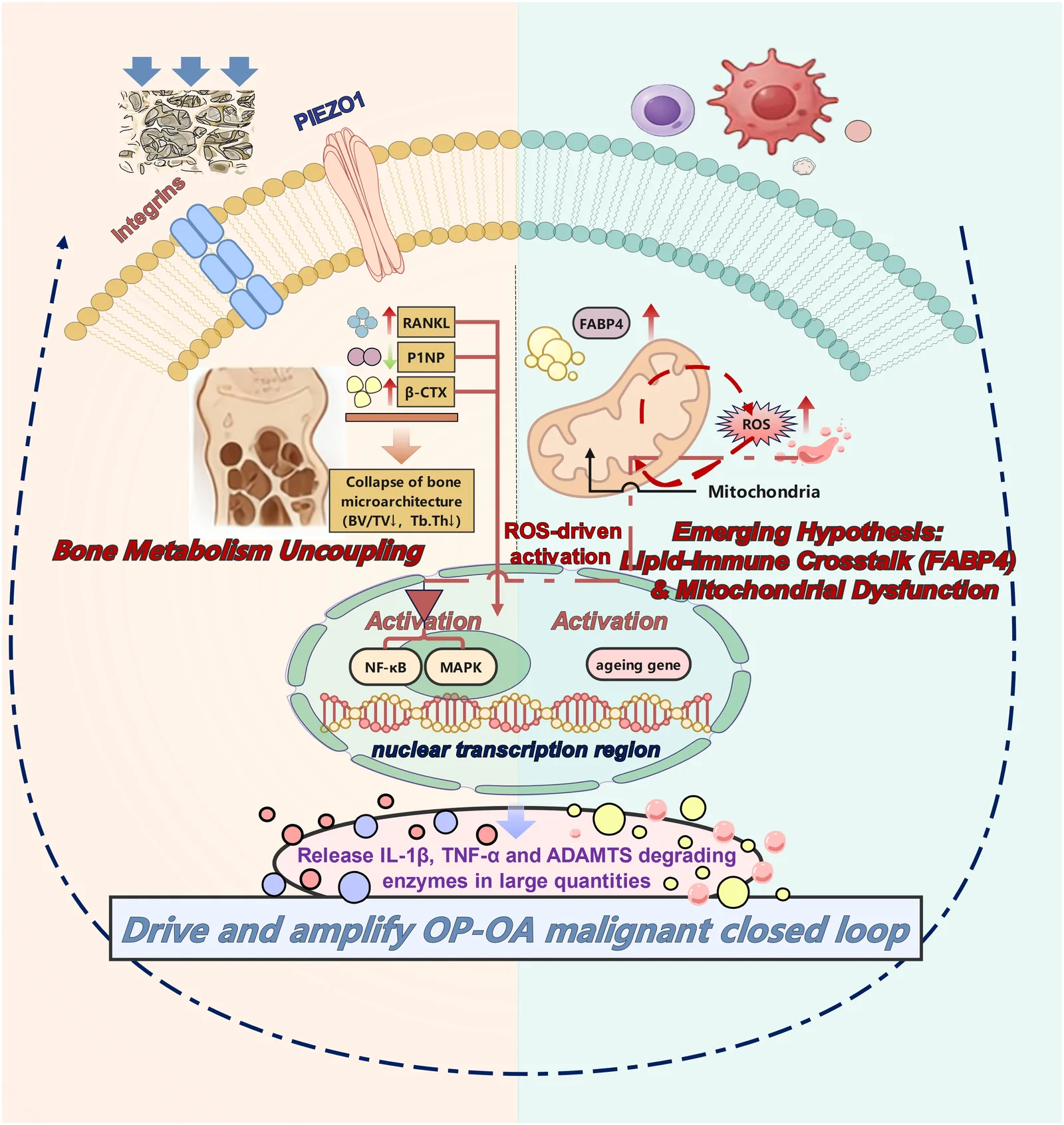
Molecular mechanisms of uncoupling of bone metabolism and “lipid-immune and mitochondrial dysfunction” driving malignant looping in OA-OP comorbid diseases.

Based on the aforementioned multicellular interaction network, emerging preclinical and clinical data suggest potential mechanistic associations between systemic bone metabolism, inflammatory pathways, and molecular markers (71, 72). According to the existing literature and clinical evidence, osteoporosis (OP) is by no means only a passive comorbidity in the course of osteoarthritis (OA); on the contrary, osteoporosis (OP) is a common disease in the treatment of OA. OP-induced abnormal bone metabolism and changes in its related markers reversely drive and accelerate the pathological progression of arthritis through multi-dimensional mechanisms such as mechanics, immunity, metabolism, and cellular aging (73). Combined with the core data systematically summarized in **Tables 2**, **3**, and the molecular pathway networks revealed in **Figure 3**, the underlying mechanisms by which OP-triggered metabolic and structural abnormalities are involved in and exacerbate arthritis progression, we propose that OP-triggered metabolic and structural abnormalities may be involved in the progression of arthritis, and can be summarized into the following four core levels.

**Table 2:** Trends and pathological significance of key inflammatory and bone metabolic markers in the arthritic microenvironment.

Type	Name	The tendency to change in arthritis	Pathological significance	References
Inflammatory markers	TNF-α, IL-6, IL-1β	Significantly higher	Directly promote synovitis, osteoclast activation, and cartilage degradation	(45, 53, 82, 90)
	CRP	Rising	It reflects the level of systemic inflammation and is positively correlated with disease activity	(45, 82)
Markers of bone metabolism	RANKL	Rising	It is the key driver of bone resorption by promoting osteoclast differentiation and activation	(60–62, 87)
	β-CTX	Rising	It reflects the enhancement of bone resorption and is associated with local subchondral bone loss and osteoclast hyperactivation in OA	(60, 85, 97, 104)
	P1NP	Decreased during active osteoarthritic subchondral bone resorption; reflects impaired osteoblast repair	P1NP reflects systemic type I collagen formation and is widely used as a bone-formation marker. Its interpretation in OA-OP should be cautious because circulating P1NP is not specific to subchondral bone and may not directly represent local osteoblast activity within the osteoarthritic joint.	(84, 97, 104)
Molecular and genetic level	FABP4	Rising	Emerging hypothesis; altered FABP4-related signaling may reflect subchondral bone marrow adipose tissue activity, but direct evidence establishing FABP4 as a causal mediator of OA-OP is currently insufficient.	(63, 88, 89, 91, 92)
	Related products of arginine metabolism (NO, polyamines)	Imbalances	It affects osteogenic differentiation, osteoclast activation, and immune regulation, and participates in osteoarthritis bone destruction	(63)

CRP, C-reactive protein; TNF-α, tumor necrosis factor-alpha; IL-6, interleukin-6; IL-1β, interleukin-1 beta; β-CTX, C-terminal telopeptide of type I collagen; P1NP, procollagen type I N-terminal propeptide; FABP4, fatty acid-binding protein 4; RANKL, receptor activator of nuclear factor-κB ligand; NO, nitric oxide; OA, osteoarthritis; OP, osteoporosis. Circulating bone-turnover markers are not specific to subchondral bone and should be interpreted together with imaging and clinical phenotype.

**Table 3:** Central mechanisms of structural and metabolic abnormality indicators driven by osteoporosis (OP) in arthritis exacerbation.

The metrics raised by OP	Change	Role in OA	References
P1NP	Lower	The inhibition of osteogenesis is not conducive to bone repair after arthritis, which may lead to the decrease of subchondral bone strength and accelerate the destruction of joint structure	(84, 97, 104)
β-CTX, RANKL	Rising	RANKL directly exacerbates periarticular bone erosion and systemic bone loss; RANKL also promotes inflammation by activating synovial fibroblasts	(50, 60–62, 85, 87, 104)
FABP4	Rising	Hypothetical bone-lipid-immune mediator: postulated to modulate M1 macrophage polarization and subchondral inflammation under joint metabolic/mechanical strain. while	(88–92)
Parameters of bone microstructure	BV/TV, Tb. Th decreased, and Tb. Sp increased	It reduces the mechanical properties of bone, makes joints more prone to deformities and fractures, and may change the biomechanical distribution of joints and accelerate cartilage wear	(74–76, 78, 79, 105, 106)
Mitochondrial function	Increased ROS and abnormal energy metabolism	Influences macrophage and osteoblast function by abnormal mitochondrial transfer, promotes inflammation and apoptosis, and drives OA progression	(93–96, 101, 102)

### 4.1 Deterioration of bone microstructural parameters leads to biomechanical collapse

The most direct destruction of osteoporosis lies in the cliff-like decline of bone quality. In the OP state, the bone microstructural parameters of the patients were significantly deteriorated, which was manifested by the significant decrease in cancellous bone volume fraction (BV/TV) and trabecular thickness (Tb. Th) in the load-bearing areas such as the femoral head and tibial plateau, while the trabecular separation (TB. SP) increased abnormally (74–76). This disintegration of bone microstructure leads to a sharp decline in the mechanical properties of the bone, which greatly reduces the carrying capacity of the joint (77). The loosening of subchondral bone “foundation” directly changes the local biomechanical distribution of the joint, making the joint more prone to micro-collapse, Varus deformity, and microfracture; thus, the abnormal wear and structural degeneration of articular cartilage are directly accelerated at the physical and mechanical level (78, 79). Therefore, the deterioration of systemic OP parameters and the disintegration of local subchondral bone microstructure are not simply unidirectional causality but the result of nested and co-amplified systemic metabolic decline and local abnormal stress networks (80, 81).

### 4.2 Uncoupling of bone metabolic markers and deterioration of the immune-bone metabolism interaction

The superposition of the inflammatory microenvironment with OP resulted in a global uncoupling of subchondral bone metabolism (shown in the left module of **Figure 3**) (82, 83). As shown in **Table 2**, the abnormalities of various inflammatory markers and markers of bone metabolism showed a high degree of pathological cooperativity. On the one hand, the n-terminal propeptide of type I procollagen (P1NP), a marker of osteogenesis, decreased significantly (84). On the other hand, the n-terminal propeptide of type I procollagen (P1NP), a marker of osteogenesis, decreased significantly. This means that the function of osteoblasts is severely inhibited, which not only hinders the repair process of bone injury after arthritis, but also leads to the continuous decline of subchondral bone strength. On the other hand, bone resorption markers (such as β-CTX, RANKL) are abnormally elevated. Highly active osteoclasts not only directly exacerbate peri-articular bone erosion and systemic bone loss but also contribute to the development of osteoclasts (85). They also release a large amount of matrix proteins such as transforming growth factor beta (TGF-β) stored in the bone matrix during intense bone resorption (86). More critically, high levels of RANKL not only drive osteoclastogenesis but also feedbackly enhance the inflammatory response of synovial and immune cells by activating synovial fibroblasts (FLS), forming a malignant interaction between the immune response and bone destruction (60–62, 87).

### 4.3 Imbalance of the “bone-lipid-immune” hub and mitochondrial dysfunction

At the systemic and local cellular energy metabolism levels, abnormal subchondral bone remodeling is frequently accompanied by altered lipid signaling, giving rise to the emerging hypothesis of a FABP4-centered ‘bone-lipid-immune’ axis. In the osteoarthritic subchondral microenvironment, recent studies on bone marrow adipose tissue (BMAT) lipolysis suggest that elevated expression of fatty acid-binding protein 4 (FABP4) may act as a potential metabolic link (88, 89). As a proposed hypothesis, subchondral FABP4 upregulation—triggered by localized mechanical overload and metabolic stress—is postulated to modulate macrophage polarization toward a pro-inflammatory phenotype and influence local bone turnover via RANKL/OPG signaling (90–92). However, whether FABP4 functions as a primary driver or a secondary biomarker in subchondral OA-OP pathogenesis remains an active hypothesis requiring direct clinical validation. In addition, mitochondrial dysfunction (such as increased reactive oxygen species and abnormal energy metabolism) also contributes to this stage (see **Figure 3** right Mitochondria module) (93–95). Abnormal mitochondrial transfer profoundly interferes with the normal physiological functions of macrophages and osteoblasts, promotes the storm of local inflammatory factors and apoptosis, and continuously promotes the progression of arthritis to an irreversible stage (96).

### 4.4 Synergistic destruction of cellular senescence

OP and arthritis highly share the underlying logic of ‘cellular senescence’ in pathogenesis (67–69). With the progression of the disease and the deterioration of the microenvironment, senescent cells accumulate in bone and joint tissues. The senescence-associated secretory phenotype (SASP) factors (such as IL-6 and various matrix metalloproteinase MMPs) secreted by these cells played an extremely harsh “double-edged sword” destructive role in the microenvironment: they both inhibited bone-forming activities of osteoblasts upward (exacerbating OP) and acted as potent disaggregate enzymes downwards, which promoted the growth of osteoblasts, and directly promoted degradation of the articular cartilage matrix (opacifying OA) (70).

In summary, from the physical collapse of bone microarchitecture (BV/TV, Tb. Th) to the biochemical uncoupling of bone metabolic indicators (elevated β-CTX, inhibited P1NP), it is important to understand the underlying mechanisms of bone microarchitecture to the synergy of the imbalance of deep metabolic hubs (FABP4, mitochondrial ROS) with cellular senescence. The high level of concurrence of these markers is not only an external manifestation of dysregulated cellular interactions, but also an important factor in the regulation of cellular senescence (97). It also forms a perfect loop of “OP bone loss-mechanical decompensation and metabolic decompensation-inflammatory cascade-more severe cartilage and bone destruction” (shown at the end of pathology at the bottom of **Figure 3**). Collectively, these findings suggest that altered bone remodeling, cellular senescence, mitochondrial stress, and inflammatory signaling may coexist and interact in OA-OP. However, the available studies do not establish that systemic osteoporosis independently causes OA progression, and several proposed molecular links remain based on preclinical or cross-disease evidence (98).

### 4.5 Cautious extrapolation of osteoimmune mechanisms from inflammatory arthritides to subchondral bone loss in OA

While rheumatoid arthritis (RA) represents a classical, extreme paradigm of severe cytokine-driven osteoclastogenesis and marginal bone erosion, direct extrapolation of RA mechanisms to osteoarthritis combined with osteoporosis (OA-OP) requires caution. RA is primarily a systemic autoimmune disorder where infiltrating adaptive immune cells (e.g., autoreactive T and B cells) directly trigger uncontrolled synovial pannus invasion and systemic RANKL hyper-expression. In contrast, OA-OP is predominantly a localized, mechanoflammatorily and metabolically driven degenerative condition. In this review, common osteoimmune mediators—such as the RANKL/RANK/OPG axis, pro-inflammatory cytokines (IL-6, TNF-α, IL-1β), and local macrophage-to-osteoclast precursor lineages—are cited not to suggest that OA is an autoimmune disease, but because they represent conserved molecular pathways of bone resorption. Crucially, these pathways operate under fundamentally distinct local microenvironmental triggers in OA: Biomechanical Strain vs. Autoimmune Activation: Subchondral RANKL upregulation in OA is predominantly driven by mechanical overload sensed via osteoblast/osteocyte mechanosensors (e.g., PIEZO1, integrins) rather than systemic autoimmune complex deposition. Localized Subchondral Niche vs. Systemic Joint Erosion: Unlike the diffuse synovial pannus erosion in RA, osteoclast activation in OA-OP is geographically restricted to subchondral bone micro-cracks, resorption lacunae, and early tidemark-penetrating micro-vessels. Recognizing these key boundary conditions allows us to cautiously leverage mechanistic insights from inflammatory arthritides to understand subchondral bone loss in OA-OP, without confounding the unique biomechanical-osteoimmune pathogenesis of OA.

Left: Bone Metabolism Uncoupling: abnormal mechanical stress (sensed via Integrins versus Piezo1 channels) with local pathological changes, resulting in decreased secretion of osteogenic markers (P1NP) and abnormally elevated bone resorption factors (RANKL, β-CTX) (99). This biochemical uncoupling directly induced the physical collapse of bone microstructure (BV/TV, Tb. Th) (100). Right: Emerging Hypothesis: Lipid-Immune Crosstalk (FABP4) & Mitochondrial Dysfunction: high expression of fatty acid-binding protein 4 (FABP4) in the microenvironment, superimposed on mitochondrial dysfunction and excessive accumulation of reactive oxygen species (ROS), which further disturbs the normal energy and metabolic homeostasis of the cell (101, 102). Bottom: Nuclear Transcription & Output: Aberrant signals from these dual pathways converge in the nuclear transcription region, activating NF-κB, the MAPK inflammatory pathway, and the ageing gene (103). This forces cells to release pro-inflammatory factors (IL-1β, TNF-α) and matrix-degrading enzymes (Adamts et al.) in large quantities, ultimately driving and exponentially amplifying the OP-OA comorbid self-perpetuating pathological feedback cycle.

The core markers that show significant abnormalities in the progression of arthritis are summarized in the table. The massive release of proinflammatory cytokines (such as TNF-α, IL-6), along with the synchronous abnormal elevation of key bone resorption drivers (such as RANKL, β-CTX), reflects the strong destructive effect of the local inflammatory microenvironment on bone remodeling, which may contribute to the development of bone resorption, marks that bone metabolism is in a severe uncoupling state.

This table illustrates the local microstructural and systemic metabolic variability triggered by osteoporosis as a core driver. Physical collapse of bone microstructural parameters (BV/TV, Tb.) The decrease vs. TB. SP increase in the load-bearing area, superimposed mitochondrial dysfunction, and the overall imbalance of “Bone-lipid-immune” metabolic hubs such as FABP4 confirm that the deterioration of systemic OP parameters has a direct accelerating effect on the destruction of joint structures.

## 5 Limitations of existing pharmacological interventions and novel strategies for comorbid treatment with targeted anti-osteoporotic interventions

At present, the clinical drug treatment for osteoarthritis (OA) mainly focuses on the two core strategies of “Relieving symptoms” and “Cartilage protection”; its action pathway is highly dependent on the inhibition of inflammatory factors and matrix degradation processes (107–110). However, in the complex pathological microenvironment of osteoarthritis and osteoporosis (OA-OP), the traditional one-way target treatment has gradually exposed significant limitations. How to break through existing treatment barriers and reshape the biomechanics and bone metabolic homeostasis in the comorbid microenvironment has become a core scientific problem to be solved in the field of orthopedics and rheumatic immunology (65).

### 5.1 Pharmacological basis and targets of conventional drug therapy for osteoarthritis

In current clinical practice, the pharmacological intervention system for OA is mainly composed of the following types of drugs, which act on different nodes of the pathological cascade (56).

The first category is non-steroidal anti-inflammatory drugs (NSAIDs, such as celecoxib, diclofenac sodium) and glucocorticoids, as the representative of the anti-inflammatory analgesic drugs, is the current clinical control of acute attacks of the first-line choice (111, 112). Its core mechanism is to inhibit the activity of cyclooxygenase-2 (COX-2) and block the synthesis of pain-causing and inflammatory mediators such as prostaglandin E2 (PGE2) (113). The glucocorticoid works through a wider range of genomic and non-genomic effects; it strongly inhibits the cascade of proinflammatory cytokines such as interleukin-1β (IL-1β), interleukin-6 (IL-6), and tumor necrosis factor-α (TNF-α), can effectively reduce synovial inflammation, eliminate joint swelling, and significantly relieve pain in the short term.

The second category is osteoarthritis-modifying drugs (DMOADS) or chondroprotectants, which typically include intra-articular sodium hyaluronate, oral glucosamine, chondroitin sulfate, etc. The biological purpose of this type of intervention is to provide an exogenous synthetic matrix to damaged chondrocytes (114–116). At the level of molecular mechanisms, it attempts to down-regulate the transcriptional and expression activities of matrix metalloproteinase (especially MMP-3, MMP-13) and ADAMTS (platelet-binding protein type I motif-containing disaggregated protein-like metalloproteinase) families in synovial and cartilage tissues, delay the enzymatic degradation of collagen II and aggrecan, the two core proteins of the hyaline cartilage mechanical buffer network (117).

### 5.2 Limitations of traditional treatment strategies and lack of consensus in diagnosis and treatment under the OA-OP comorbidity model

**Figure 4:**
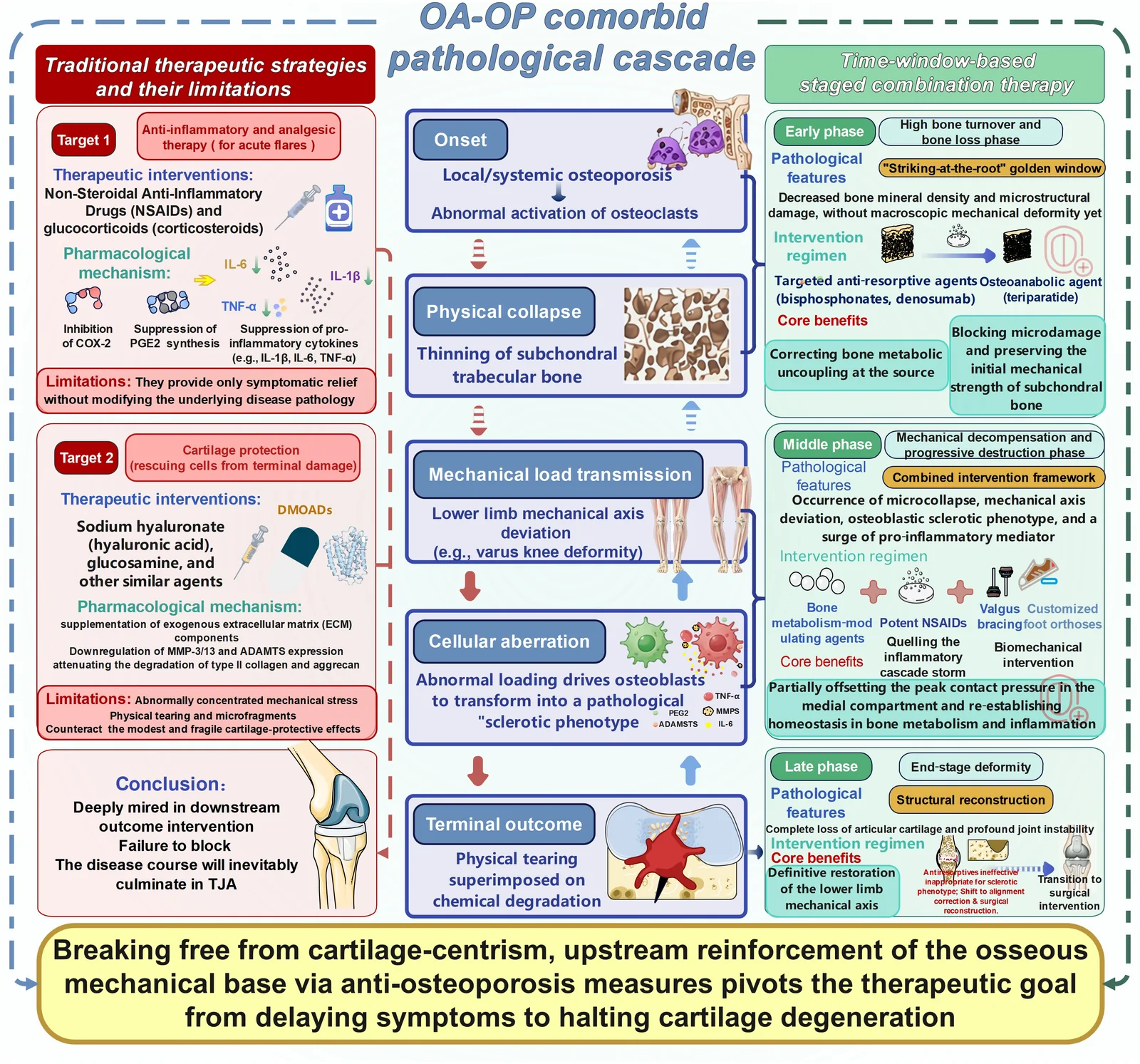
Comparison of OA-OP comorbid pathological cascades and “time window”-based staged combination therapy strategies.

Although the above strategies have achieved some efficacy in the management of early symptoms of OA, from a mechano-biological global view of the “Osteoporotic-arthritis comorbidity”, it is clear that the aforementioned strategies are effective in the management of early symptoms of OA; existing interventions have insurmountable limitations (as shown in the left module of **Figure 4**) (118). Its fundamental defect lies in its deep-rooted “Cartilage-centrism” research perspective, which seriously ignores the key role of subchondral bone as the “Mechanical foundation” of joints. Adult articular cartilage is located in an extremely isolated microenvironment with no blood vessels, no nerves, and low cellular metabolism (119–121). Its histological characteristics determine that once the cartilage is physically stripped, it will be highly non-renewable.

In OA-OP comorbid patients, traditional chondroprotective or anti-inflammatory treatments alone intervene only on the “downstream outcome” of the disease (i.e., the attempt to rescue the already irreversible chondrocytes), and the treatment of the disease is not sufficient to prevent the development of the disease, failure to block the real “upstream etiology”—the thinning of subchondral trabeculae and collapse of microstructure caused by local or systemic osteoporosis, and subsequent deviation of the alignment of the lower limbs (such as Genu Varus Deformity) (106). When the abnormal mechanical stress concentration persists, the resulting physical tearing and microfragmentation are sufficient to counteract any weak pharmacological chondroprotective effect (122). This leaves the patient mired in downstream outcome interventions that eventually lead to total joint replacement (Tja). In addition, there is currently no unified clinical diagnosis and treatment guideline or consensus for OA-OP co-morbidity (123). In most cases, clinicians treat OA with OP as two independent diseases in a split-type manner, and the treatment of OA-OP is still limited. The lack of comprehensive intervention programs targeting the comorbid cascade network (such as the “Bone-fat-immune” hub and the “Bone-cartilage” crosstalk) has led to many patients taking drugs regularly, the course of the disease is still inexorable to the end stage, and finally only relies on artificial joint replacement (124, 125).

### 5.3 Principles of combined treatment by stages and choice of intervention time window for OA-OP comorbid diseases

In view of the complexity of comorbid mechanisms, the current academic treatment of OA-OP comorbid diseases is gradually shifting to the principle of comprehensive intervention with “Mechano-biological” bidirectional regulation, and the development of new strategies for the treatment of OA-OP comorbid diseases, as shown in the pathological cascade and the intervention strategy on the right in **Figure 4** (126). The core is to break the previous single-drug model, emphasizing staging and combination therapy according to the “time window” of disease progression (127).

The “time window” for intervention is crucial in timing and planning. First, in the early phase of comorbidity (characterized by high bone turnover and active bone loss), osteoclasts in the subchondral bone are hyperactivated, causing microstructural trabecular thinning and local bone density reduction before macroscopic cartilage erosion occurs. This period represents the ‘Golden Window’ for targeted antiresorptive intervention (e.g., bisphosphonates or denosumab) or osteoanabolic therapy (e.g., teriparatide) to preserve subchondral structural integrity and prevent stress concentration (127–129).Crucially, the clinical efficacy of anti-osteoporotic therapy is strictly dependent on matching the drug mechanism with the prevailing subchondral bone turnover phenotype (130). As OA progresses into late stages, subchondral bone undergoes a temporal biphasic transition into a low-turnover, sclerotic phenotype. In this phase, aberrant osteoblastic hyper-mineralization produces dense but pathologically brittle sclerotic bone with diminished elasticity, while osteoclast activity is relatively suppressed. Administering potent antiresorptive agents during this sclerotic phase is not only ineffective but clinically inappropriate. Further suppressing bone remodeling in a low-turnover environment hampers the physiological clearance of subchondral micro-cracks, exacerbates tissue brittleness, and fails to mitigate mechanical stress transferred to the overlying cartilage.

This phenotypic mismatch provides a compelling mechanistic explanation for why randomized controlled trials (RCTs) and systematic meta-analyses (e.g., Zhang et al., 2022) testing bisphosphonates in broad, unselected OA populations have failed to demonstrate consistent relief of knee pain or reduction in bone marrow lesions (BMLs) (131). Unselected cohorts predominantly comprise patients in middle-to-late sclerotic stages. Therefore, to make the proposed time-window framework clinically actionable, anti-osteoporotic regimens must be guided by diagnostic phenotyping—reserving antiresorptives strictly for high-turnover early bone loss while shifting toward alignment correction, anti-inflammatory measures, or surgical reconstruction in low-turnover sclerotic stages.

Second, in the middle stage of comorbid diseases (mechanical decompensation and progressive destruction), the subchondral bone has been slightly collapsed, and the biological force line of the lower limb begins to shift; abnormal loads drive osteoblasts to convert to a pathological “sclerosing phenotype” and release a plethora of pro-inflammatory mediators (132). At this point, anti-osteoporosis drugs alone are often less effective (which is why some RCT studies of bisphosphonates in OA have been controversial). Treatment options at this stage must shift to “co-intervention”: not only must bone metabolism continue to be modulated, but NSAIDs must be introduced forcefully to quell the “inflammatory cascade” originating in the subchondral bone, which is a critical component of bone metabolism (133). Biomechanical interventions, such as valgus braces and custom insoles, were used to partially offset the peak contact pressure in the medial compartment.

Third, in the late stage of comorbid diseases (full-thickness cartilage denudation and end-stage deformity), the joint structure is completely unstable, and the cartilage is completely lost (134). At this time, pharmacological intervention cannot reverse the structural damage, and the treatment plan can only be converted to surgical intervention based on osteotomy or artificial joint replacement to completely reconstruct the mechanical axis and joint surface of the lower limb (135).

To facilitate clinical translation and provide actionable guidance for practitioners, we have summarized the explicit diagnostic benchmarks and stage-specific intervention strategies in **Table 4**. By integrating DXA bone mineral density (T-score), specific subchondral MRI features (e.g., bone marrow lesions, micro-cracks, cartilage loss), and macroscopic biomechanical alignment indicators (varus angle), this ‘Time Window’ classification enables clinicians to precisely categorize OA-OP patients and implement tailored combined therapeutic regimens across early, middle, and late phases. To provide a comprehensive synthesis of the translational and clinical landscape, we have systematically summarized the key empirical evidence, therapeutic outcomes, levels of evidence, and major limitations of principal bone-targeting agents in Osteoarthritis in **Table 5**.

**Table 4:** Stage-specific clinical/imaging diagnostic benchmarks and therapeutic strategies for OA-OP comorbidities based on the “time window” framework.

Phase	Characteristics of pathological mechanism	Clinical and imaging diagnostic criteria (BMD, MRI, mechanical axis)	Primary therapeutic targets	Recommended intervention regimen
Early Phase	Abnormal activation of osteoclasts, thinning of trabeculae, microdamage of subchondral bone and uncoupling of bone metabolism.	**BMD(DXA):** T-score –1.5 to –2.5 (Osteopenia to early OP) **MRI/CT:** Subchondral BMLs, trabecular bone volume (BV/TV) ↓, intact cartilage thickness (K-L grade I) **Alignment:** Normal or mild alignment deviation (Varus angle<3^∘^)	**“Striking-at-the-root”**: Correcting subchondral uncoupling, preserving trabecular microarchitecture, blocking upstream mechano-inflammation.	**Anti-resorptive agents:** Bisphosphonates (Zoledronic acid), Denosumab **Osteoanabolic agents:** Teriparatide (PTH1-34), Romosozumab (anti-Sclerostin) ***Core Goal:*** Stabilize subchondral mechanical base before macroscopic cartilage erosion.
Middle Phase	Subchondral micro-collapse, osteoblastic sclerotic phenotype conversion, surge of pro-inflammatory SASP and matrix degrading enzymes.	**BMD(DXA):** Localized/systemic OP (*T-score*≤–2.5), focal subchondral bone sclerosis coexisting with regional osteopenia **MRI/CT:** Subchondral micro-cracks, erosion lacunae, tidemark-penetrating vessels, joint space narrowing (K-L grade II–III) **Alignment:** Progressive force line shift (Varus angle 3^∘^ --8^∘^)	**“Combined Multi-Modal Intervention”**: Re-establishing bone-cartilage homeostasis, quelling inflammatory cascade, rebalancing peak contact pressure.	**Targeted Pharmacotherapy:** Bone metabolism modulators + Potent NSAIDs/COX-2 inhibitors **Biomechanical Intervention:** Valgus knee bracing, custom lateral wedge insoles ***Core Goal:*** Offset compartment overload and silence subchondral inflammatory signaling.
Late Phase	Irreversible cartilage loss, extensive subchondral plate collapse, profound joint instability, fixed lower limb mechanical axis malalignment.	**BMD(DXA):** Severe osteoporosis (T-score < –3.0) **MRI/X-ray:** Full-thickness cartilage denudation, extensive osteophytes, severe subchondral bone sclerosis/cysts (K-L grade IV) **Alignment:** Severe fixed deformity (Varus angle > 8°)	**“Structural Reconstruction & Alignment Restoration”**: Surgical correction of mechanical alignment and joint surface restoration.	**Surgical Intervention:** High Tibial Osteotomy (HTO) or Total Knee Arthroplasty (TKA) **Perioperative Management:** Continued anti-osteoporosis therapy to optimize peri-implant bone quality and fixative stability.

BMD, Bone Mineral Density; BMLs, Bone Marrow Lesions; BV/TV, Bone Volume to Total Volume; DXA, Dual-energy X-ray Absorptiometry; HTO, High Tibial Osteotomy; K-L, Kellgren-Lawrence grading system; NSAIDs, Non-Steroidal Anti-Inflammatory Drugs; OP, Osteoporosis; TKA, Total Knee Arthroplasty.

Bold values indicate the key diagnostic and therapeutic criteria and the principal clinical findings emphasized by the authors.

**Table 5:** Translational and clinical evidence of principal bone-targeting agents in OA.

Drug class & agent	Primary study types & models	Target OA phenotype & OP status	Key clinical/preclinical outcomes	Level of evidence	Major limitations & clinical gaps
Bisphosphonates (Zoledronic Acid, Risedronate)	Meta-analyses of RCTs (Zhang et al., 2022); Animal models	Unselected knee OA; Variable OP status	Reduced bone marrow lesions (BMLs) in preclinical models; **Failed to show consistent pain or BML improvement in clinical meta-analyses**	High (Meta-analysis)/Preclinical	Lack of patient phenotype stratification; Ineffective in late-stage low-turnover sclerotic phenotypes.
RANKL Inhibitor (Denosumab)	Phase II RCTs; Preclinical OVX-OA models	High-turnover early OA; Postmenopausal OP	Preserved subchondral trabecular microarchitecture; Mixed results on cartilage loss and pain score	Moderate (Phase II)	Limited large-scale long-term OA RCTs; Risk of rebound bone resorption upon discontinuation.
PTH Analog (Teriparatide)	Preclinical rodent models; Observational cohorts	Osteoporotic OA; Low bone mass	Promoted chondrocytic anabolic repair and subchondral trabecular bone formation	Low/Preclinical	Potential risk of exacerbating subchondral osteophytes if over-administered.
Anti-Sclerostin (Romosozumab)	Preclinical models; Target trial emulation studies	Secondary OP with joint degeneration	Dual anabolic and anti-resorptive effects; Improved microstructural density	Low (Preclinical/Observational)	High cost, potential cardiovascular risk profile, lack of randomized controlled trials specifically in OA.

Bold values indicate the key diagnostic and therapeutic criteria and the principal clinical findings emphasized by the authors.

### 5.4 strategy transformation from cartilage protection to mechanical source intervention

Based on the aforementioned clinical dilemma and the limitations of existing interventions, this article aims to address a central scientific question: How to break through the physiological limitation that adult articular cartilage is highly non-regenerative before the irreversible destruction of OA occurs, and how to prevent the damage of OA, find an upstream therapeutic pathway that blocks both microinflammatory degradation and macromechanical imbalance?

The innovation of this article is to go beyond the traditional “Chondrocentrism” and explicitly propose a new treatment strategy of “Anti-osteoporosis to slow down the progression of arthritis” (see the core concept at the bottom of **Figure 4**). We have moved the therapeutic target forward as a whole, and the dynamic changes of bone mass and biomechanical properties caused by osteoporosis have become the core pathological regulation hub throughout the whole course of OA deterioration (136). By intervening within a potential early high-turnover time window offers a biologically plausible hypothesis to stabilize the mechanical base and slow progressive joint structural degeneration. Nevertheless, translating this biological rationale into clinical efficacy requires rigorous validation through phenotypically stratified trials rather than unselected clinical application.

(left) traditional therapeutic strategies and their limitations: traditional anti-inflammatory analgesia (target 1) and chondroprotective (target 2) interventions are mired in ‘chondrocentrism’ and can only provide symptom relief downstream. Due to the inability to counteract the microfragments and structural physical tears caused by abnormal mechanical stresses, its efficacy eventually inevitably leads to failure and total joint replacement (Tja).(middle row) OA-OP comorbid pathological cascade: revealed from local/systemic osteoporosis (abnormal activation of osteoclasts), to physical collapse (thinning of subchondral trabeculae), mechanical conduction abnormalities (deviation of lower limb force lines), cell phenotypic aberrations (abnormal loading drives osteoblastic sclerosis), and finally to the final evolutionary trajectory of physical and chemical double destruction.(right)”Time window”-based staged combination therapy: emphasis on precise intervention based on pathological stage. The early stage (high bone turnover period) is the “Golden Window” for targeting anti-osteoporotic agents, which aim to block microdamage at the source and stabilize the mechanical base. In the middle stage (progressive destruction stage), bone metabolic modulators, potent NSAIDs, and biomechanical braces should be combined to reestablish metabolic and inflammatory homeostasis and counteract abnormal peak pressures. In the late stage (end-stage deformity), only surgical reconstruction is needed.(bottom) therapeutic paradigm shift: emphasis on moving therapeutic targets forward and strengthening the bony mechanical substrate upstream through anti-osteoporosis measures to achieve a fundamental shift from simply ‘delaying symptoms’ to a substantial ‘arresting cartilage degeneration’. Solid arrow: established component mechanism; Dashed arrow: proposed therapeutic consequence;

### 5.5 Theoretical biological rationale for targeting subchondral remodeling in OA

**Figure 5:**
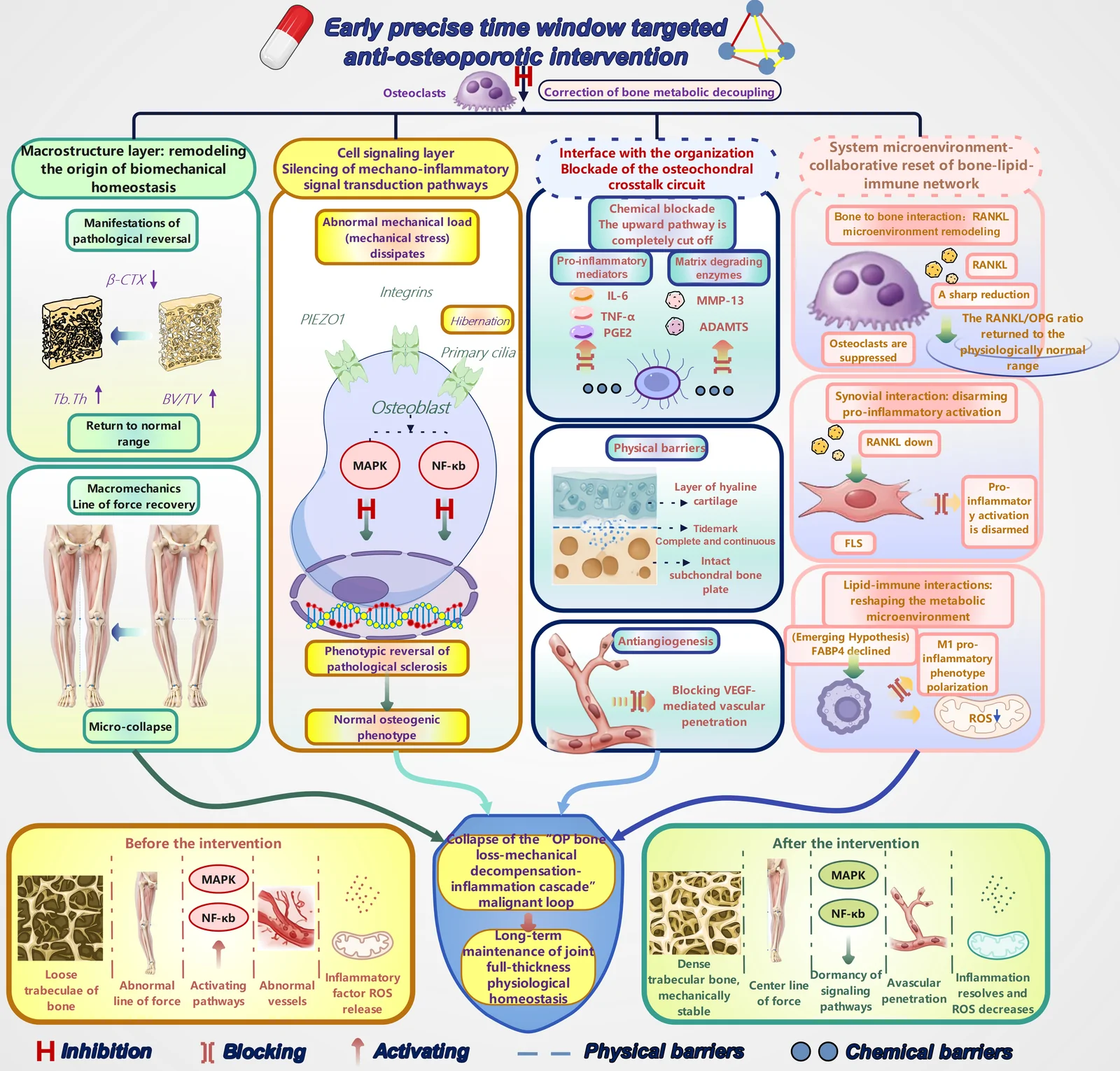
Multi-dimensional mechanistic framework of early precision time-window anti-osteoporotic intervention in reshaping OA-OP subchondral microenvironment homeostasis.

Establishing anti-osteoporosis as an upstream breakthrough for co-morbidity treatment is not only highly prospective in theory, but also shows strong biological feasibility at the level of molecular mechanism and mechanical delivery network (137). If early, staged, and targeted anti-osteoporosis interventions can be implemented, the joint microenvironment will present a “mechano-biological” double homeostatic reversal in the following four dimensions, as shown in the global mechanism map of **Figure 5**.

#### 5.5.1 Headstream remodeling of biomechanical homeostasis: physical stabilization of subchondral bone microstructure

As shown in **Figure 5**, “Macrostructure layer,” early intervention with anti-osteoclast drugs that inhibit osteoclast activity directly corrects the uncoupling of bone metabolism in subchondral bone. The cancellous bone volume fraction (BV/TV) and trabecular thickness (Tb. Th) in load-bearing areas such as the tibial plateau were maintained or restored to normal levels as the levels of bone resorption markers such as β-CTX decreased and the bone network was gradually repaired. The physical stability of the microstructure may reduce the risk of the diffuse micro-collapse of the subchondral bone that cannot compensate for the physiological load, from the macroscopic physical source, the medial shift of the force line of the lower limb (genu varus deformity) caused by the disintegration of the microstructure is blocked, so that the cartilage is protected from the concentrated stress stripping beyond the physiological threshold (138).

#### 5.5.2 Silencing of mechano-inflammatory signal transduction pathways: reversal of the pathological phenotype of osteoblasts

The subchondral bone serves as a bi-directional transformation hub for “mechanical signaling-biological effects,” as illustrated by the “cell signaling layer” path in **Figure 5**. After the pine has gained control, the load-bearing mechanical base is restored to stability, and the subchondral bone is stabilized, the abnormally elevated mechanical loading signal then dissipates (139). The PIEZO1 ion channels, integrin adhesion network, and primary ciliary structures on the surface of osteoblasts are no longer overactivated after losing the drive of overloaded physical stimuli (140, 141). This will directly lead to intracellular MAPK and NF-κB and other stress- and inflammation-related pathways into a dormant or silenced state, effectively inhibiting the malignant transformation of osteoblasts to a pathological “sclerosing phenotype”.

#### 5.5.3 blockade of the osteochondral crosstalk circuit: potential attenuation of ascending degradation signals

As osteoblasts return to a normal phenotype, their cellular secretion profile is also globally corrected. Pro-inflammatory mediators such as IL-6, TNF-α, and PGE2 are no longer released in large quantities in the microenvironment. More importantly, the overexpression of matrix-degrading proteases such as MMP-13 and Adamts family is may be attenuated. In addition, as shown by the physical and chemical barriers constructed by the “Tissue interface layer” in **Figure 5**, VEGF-mediated penetration of destructive blood vessels through the tidal line is blocked because the subchondral bone plate no longer produces microfissures due to abnormal stress. This dual effect is equivalent to substantially attenuating the physical and chemical pathways through which inflammatory factors and matrix-degrading enzymes penetrate into the avascular hyaline cartilage layer from the bottom, type II collagen and aggrecan are effectively protected from degradation.

#### 5.5.4 Synergistic reset of the bone-lipid-immune network: rebalancing of the local versus systemic metabolic microenvironment

Targeted anti-osteoporotic therapy not only acts on the bone itself but also profoundly resets the cellular multidimensional interaction network in the microenvironment. Effective inhibition of osteoclastic activity will significantly down-regulate the RANKL level in the local microenvironment and restore the RANKL/OPG ratio to a normal range (60, 142, 143). This not only directly reduced bone resorption activity, but also abolished the pro-inflammatory activation effect of high levels of RANKL on synovial fibroblasts (FLS) (144). Moreover, targeted stabilization of subchondral bone density may help dampen abnormal lipid metabolic signals, including the hypothetical FABP4-mediated ‘bone-lipid-immune’ cascade. By attenuating localized metabolic strain and suppressing BMAT lipolysis, early intervention could potentially reduce pro-inflammatory macrophage activation and reactive oxygen species (ROS) accumulation in the subchondral niche, thereby interrupting the proposed metabolic closed-loop of OA progression.

Early targeted inhibition of excessive osteoclastic bone resorption and correction of subchondral bone metabolic uncoupling are proposed to achieve microenvironmental reversal across four synergistic layers: Macrostructure layer (Established Evidence): Decreased bone resorption activity (β-CTX↓) preserves cancellous microarchitecture (Tb.Th↑, BV/TV↑), physically stabilizing subchondral bone trabeculae to mitigate local micro-collapse. Cell signaling layer (Established/Validated Pathways): Attenuation of localized mechanical overload dampens mechanosensing receptor activation (PIEZO1 channels, integrins, primary cilia) on osteoblasts, leading to the downstream silencing of mechanoflammatory MAPK and NF-κB pathways and suppressing pathological osteoblastic sclerotic transdifferentiation. Interface with the organization (Validated Interface Barrier): Chemically blocks the upward diffusion of subchondral pro-inflammatory cytokines (IL-6, TNF-α, PGE2) and matrix-degrading enzymes (MMP-13, ADAMTS family) into the articular cartilage. Physically maintains the structural integrity of the tidemark and inhibits VEGF-driven pathological Type H vessel invasion. System microenvironment layer (Emerging Hypothesis): Synergistically resets the osteoimmune network. Downregulation of local RANKL levels disarms synovial fibroblast (FLS) pro-inflammatory activation. [Emerging Hypothesis] Decreased FABP4 expression within subchondral marrow adipose tissue (BMAT) is postulated to attenuate M1 macrophage polarization and reduce mitochondrial ROS accumulation, disrupting the localized metabolic-inflammatory loop. suppression or physical/chemical barrier blockades. Solid arrow: established component mechanism; Dashed arrow: proposed therapeutic consequence;

## 6 Discussion

Comparison with Existing Literature and Unique Conceptual Contributions:

To clarify the novel niche of this review within the rapidly evolving landscape of subchondral bone research, it is informative to contrast our framework with pivotal publications in this field, such as the work by Hu et al. (2021) on subchondral microenvironments in OA pain and the recent study by Zhang et al. (2026) on cell fate plasticity in OP-OA comorbidities (summarized systematically in **Table 6**) (119, 145). While Hu et al. laid the groundwork for understanding subchondral BMLs and neurovascular invasion in primary OA, and Zhang et al. provided an extensive mapping of stem cell lineage bias and epigenetic reprogramming in comorbid states, the present review delivers three novel theoretical and translational advances: First, we establish a conceptual or mechanistically plausible linkage between macroscopic biomechanical force line deviation (e.g., knee varus deformity) and microscopic subchondral osteoimmune reprogramming. We demonstrate how subchondral trabecular micro-collapse under systemic/local osteoporosis initiates stress concentration, activating PIEZO1/integrin mechanosensors on osteoblasts and forcing a shift toward a pathological ‘sclerotic phenotype’ that fuels ascending osteochondral degradation. Second, we refine the molecular crosstalk by highlighting the ‘Bone-Lipid-Immune’ axis centered on FABP4-mediated macrophage M1 polarization and mitochondrial ROS production, alongside clarifying that tidemark-penetrating Type H vessels serve as direct physical and humoral channels transporting subchondral elevated inflammatory cascade mediators into the hyaline cartilage. Third, and most importantly, we move beyond descriptive summary to propose a ‘Time-Window-Based Staged Biomechanical-Osteoimmune Intervention Model’. By establishing explicit quantitative diagnostic benchmarks (integrating BMD T-scores, MRI bone marrow lesions, and radiological varus angles in **Table 4**), this framework provides clinicians with a concrete roadmap to pivot from traditional ‘cartilage-centric’ symptom management to early, upstream reinforcement of the osseous mechanical base—effectively collapsing the malignant OP-OA closed loop before terminal cartilage destruction occurs.

**Table 6:** Conceptual comparison and unique contributions of the present review versus key published overviews on subchondral bone and OA-OP comorbidities.

Analytical Dimension	Hu et al. (Bone Res, 2021) (119)	Zhang et al. (IJMS, 2026) (145)	Present Review
Primary Scope & Focus	Subchondral bone microenvironment, BMLs, angiogenesis, and nerve innervation in isolated OA and pain.	Cell fate plasticity (BMSCs, chondrocytes, osteoimmune cells) driven by subchondral bone in OP-OA comorbidity.	Integrated biomechanical-osteoimmune crosstalk and temporal staged intervention paradigm in OA-OP comorbidity.
Biomechanical Integration	Discusses local subchondral stress and microstructural changes (rod/plate ratios).	Reviews mechanical load sensing and matrix stiffness regulation of stem cell fate.	Bridging macroscopic lower-limb force line shifts (genu varus deformity) with microscopic mechanotransduction (PIEZO1/Integrin/MAPK) and subchondral micro-collapse.
Cellular & Molecular Depth	Focuses on TGF-$\beta$, VEGF, Type H vessels, and NGF-mediated pain signaling.	Focuses on epigenetics, BMSC adipogenic lineage bias, SASP, and developmental signaling networks (Wnt, Hedgehog, TGF-$\beta$).	Identifies the “Bone-Lipid-Immune” hub (FABP4/M1 polarization/ROS) and tidemark-penetrating Type H vessels as physical/humoral conduits.
Therapeutic Logic	Reviews pharmacological targets against subchondral bone remodeling, angiogenesis, and pain.	Discusses phenotype-based stratification and general combined interventions (anti-resorptive, anabolic, cartilage protection).	“Cartilage-Centrism” paradigm shift $\rightarrow$ Upstream reinforcement of the osseous mechanical base.
Clinical Staging & Translability	Does not propose a temporally staged intervention model.	Mentions stratified management but lacks explicit diagnostic benchmarks or time-window regimens.	Explicit “Time Window” framework (**Table 4**) mapping Early, Middle, and Late phases to BMD T-scores, MRI BMLs, Varus angles, and multi-modal regimens.

The progression of cartilage degeneration in osteoarthritis (OA) is increasingly viewed not merely as an isolated local event, but rather as a complex process potentially intertwined with the decompensation of subchondral bone mechanics typically associated with osteoporosis (OP) (1, 119). Current literature suggests a multidimensional regulatory pattern in this OA-OP comorbid process. From a temporal perspective, observations indicate a potential sequential cascade where bone metabolic uncoupling may precede and exacerbate mechanical decompensation, eventually contributing to terminal cartilage degeneration. In this context, the structural compromise of the subchondral bone microstructure is widely discussed as a potential early driver of OA joint destruction.

Exploring the underlying mechano-biological signal transduction provides further context for these clinical observations. Current evidence indicates that abnormal mechanical stress may engage mechanoreceptors on osteoblasts, such as PIEZO1, integrins, and primary cilia, potentially activating the MAPK and NF-κB inflammatory pathways. This activation is hypothesized to promote a shift toward a pathological sclerotic phenotype, which could lead to the subsequent release of pro-inflammatory mediators and matrix-degrading enzymes into the avascular cartilage layer. Furthermore, discussions surrounding osteo-cartilage crosstalk and the osteo-lipo-immune network highlight the imbalance of the RANKL/RANK/OPG axis and FABP4-mediated macrophage M1 polarization as key molecular features within the comorbid microenvironment.

These mechanistic observations offer a valuable lens through which to view current conventional treatment strategies, which have predominantly remained “cartilage-centric”. Standard interventions, such as anti-inflammatory analgesics and chondroprotective agents, typically address the downstream outcomes or symptomatic manifestations of the disease. Given the inherently limited regenerative capacity of adult articular cartilage, these approaches may face intrinsic challenges in mitigating the upstream pathogenic drivers originating from subchondral bone mechanical decompensation. Consequently, such downstream interventions often show limited success in reversing end-stage disease progression.

Given the profound cellular heterogeneity and microstructural complexity of the subchondral bone microenvironment, unraveling early OA-OP comorbidity mechanisms requires high-resolution analytical tools. Emerging advanced methodologies, particularly Single-cell RNA sequencing (scRNA-seq) and Spatial Transcriptomics (ST), offer unprecedented opportunities to decipher these localized intercellular dialogues. By leveraging scRNA-seq, future studies can map the high-dimensional transcriptional landscapes of subchondral niche cells, resolving distinct osteoblast subsets (e.g., mechanical overload-sensitive osteoprogenitors versus dysfunctional pre-sclerotic osteoblasts) and heterogeneous macrophage subpopulations (e.g., M1-like pro-inflammatory macrophages, tissue-resident macrophages, and osteoclast-like pre-fusion lineages) during early bone turnover uncoupling. Crucially, coupling scRNA-seq with Spatial Transcriptomics preserves the native microarchitectural context, enabling *in situ* visualization of specific osteoblast-macrophage interactions at critical micro-pathological niches, such as subchondral trabecular micro-cracks, early resorption lacunae, and tidemark-penetrating Type H vessels. This spatial multi-omics framework will allow researchers to P1NPoint spatially restricted ligand-receptor communications (e.g., CSF1-CSF1R, RANKL-RANK, IL-6/STAT3, and FABP4-mediated metabolic axes), thereby identifying precise cell-type-specific targets for early time-window interventions before irreversible joint structural collapse occurs.

Furthermore, recognizing the temporal transition from an early high-turnover resorptive state to a late low-turnover sclerotic state is paramount for clinical translation. Antiresorptive therapies must not be applied indiscriminately across all OA stages. In low-turnover sclerotic subchondral bone, antiresorptive administration risks frozen bone turnover and impaired micro-damage repair. Precision phenotyping—utilizing systemic bone turnover markers (e.g., serum β-CTX, P1NP), high-resolution quantitative CT (HR-pQCT), and dynamic contrast-enhanced MRI—is essential to stratify patients who possess an active osteoclastic subchondral profile suitable for early antiresorptive interception (104, 105).

In light of these clinical challenges, exploring early, staged, and targeted anti-osteoporosis interventions presents an alternative upstream perspective for addressing joint structural degeneration. The theoretical framework for this approach encompasses four potential synergistic dimensions: mechanically stabilizing the subchondral bone to potentially mitigate micro-collapse and force line deviation; modulating mechano-inflammatory signaling to influence osteoblast phenotypic changes; isolating ascending degradation signals to preserve cartilage matrix integrity; and systemic remodeling of the bone-lipid-immune network. Rather than establishing definitive cures, this concept broadens the traditional scope of cartilage protection and invites further investigation into targeted upstream strategies for OA-OP comorbidities. Future research could focus on identifying specific patient subgroups—particularly those exhibiting high bone turnover and early bone loss—through precise imaging and metabolic assessments. Investigating individualized interventions within optimal time windows will be crucial to exploring whether such strategies can successfully transition from merely managing symptoms to meaningfully altering the overall disease trajectory.

## Limitations

This narrative synthesis has important limitations. OA is heterogeneous, and the epidemiological relationship between radiographic OA and systemic BMD is inconsistent. Many mechanistic studies are preclinical, cross-sectional, or derived from RA and therefore cannot establish OA-specific causality. Clinical evidence for bisphosphonates and other bone-targeting agents in knee OA remains inconsistent and controversial, as high-quality systematic reviews (e.g., Zhang et al., 2022) have confirmed a lack of pain or structural benefit in unselected cohorts. Thus, the proposed ‘golden window’ framework currently serves as a prospective therapeutic model requiring future validation in phenotypically stratified clinical trials.; the proposed time windows and alignment thresholds require prospective validation. Future work should use longitudinal multimodal imaging, bone-turnover markers, and single-cell/spatial transcriptomics to define treatment-responsive phenotypes.
